# Predictors of perceived value of multi-cancer early detection testing: a national PRECEDE–PROCEED framework study

**DOI:** 10.1007/s10552-026-02242-0

**Published:** 2026-09-20

**Authors:** Duxiao Hao, Chuan-Jian Zhong, Safa Elkefi

**Affiliations:** 1 School of Systems Science and Industrial Engineering, Binghamton University, State University of New York, Binghamton, NY 13902 USA; 2 Chemistry, Binghamton University, State University of New York, Binghamton, NY USA

## Abstract

**Background:**

Multi-cancer early detection (MCED) tests present a paradigm transform in cancer screening by detecting multiple cancer types from one blood sample. While studies have shown high levels of public interest in MCED screening, little is known about factors shaping individuals’ perception of the emerging technology. Guided by the PRECEDE–PROCEED framework, we aimed to identify factors associated with the perception of value of MCED testing among American adults.

**Methods:**

We conducted a cross-sectional secondary analysis of the Health Information National Trends Survey cycle 7 (HINTS 7) to identify factors associated with the perceived value of MCED screening. After removing respondents with missing outcome data, our final sample size was 6,728. Using the PRECEDE–PROCEED framework, candidate predictors were grouped into predisposing, reinforcing, and enabling factors. We used descriptive, survey-weighted analyses, spearman correlation analyses, survey-weighted multivariable logistic regression, and supervised machine learning analyses (logistic regression and random forest) to explore the associations.

**Results:**

Overall, 74.9% of the sample perceived MCED testing as very valuable. Trust-related domains showed the strongest associations with the outcome, including trust in physicians (OR = 1.89), trust in science (OR = 1.80), and trust in the healthcare system (OR = 1.64). Cancer information seeking (OR = 1.59), online health information use (OR = 1.63), smartphone access (OR = 1.54), cancer fatalistic beliefs (OR = 1.52), trust in government (OR = 1.50), knowledge of hepatitis B as a cancer risk factor (OR = 1.48), and family history of cancer (OR = 1.45) were also significantly associated with higher perceived value. Machine learning analyses demonstrated consistent predictor rankings, highlighting trust and health information engagement as the most influential determinants.

**Conclusions:**

The value of MCED screening was largely driven by trust, cancer information seeking, and access to healthcare and health information resources. Our study highlights the efficacy of the PRECEDE–PROCEED approach to studying factors associated with complex health outcomes. Future implementation strategies should prioritize equitable communication, strengthen public trust, and improve access to reliable health information to facilitate informed decision-making regarding MCED testing.

## Introduction

### Background

Multi-cancer early detection (MCED) tests represent a transformative paradigm in oncological screening, emerging from recent technological breakthroughs in genomics and liquid biopsy [1]. Launched to identify cancer-associated signals, including circulating tumor DNA (ctDNA) from a routine blood draw, these tests can reportedly simultaneously screen for several cancer types, some without a standard screening test available [2]. This is in contrast to more traditional screening methods, which are typically organ-specific or type-based, like a mammogram for breast cancer or low-dose computed tomography (LDCT) for lung cancer. As a result, standardized screening is frequently limited by age and risk factor/integration-based eligibility criteria, so that a thorough screening pipeline is an expensive and logistically challenging proposition requiring multiple separate diagnostic tests [3]. The advantages of MCED tests could be substantial, with the multi-cancer early detection (MCED) tests being regarded as a revolutionary shift in oncological screening testing, possible only through recent tech advances in genomics and liquid biopsy [1, 4]. Such “liquid biopsies” will identify cancer-specific signals (e.g., circulating tumor DNA [ctDNA]) from a standard blood draw and, in many cases, will test for multiple tumors, including some without an extant screening strategy available [2, 5]. This differs dramatically from traditional screening approaches, whereby eligibility is determined by the target organ (e.g., mammography for breast cancer; low-dose computed tomography [LDCT] for lung cancer) [3]. Despite the potential benefit of MCED testing, several aspects of its clinical application in practice remain debated. While a significant number of studies have proven the test’s sensitivity in screening multiple cancer types, concerns have been raised regarding false-positive results, overdiagnosis, additional diagnostic tests, affordability, and the best treatment options when the tissue of origin or the appropriate follow-up is unclear. Moreover, the evidence for long-term clinical benefit from population screening, such as mortality reduction, is still emerging. Therefore, despite the many advantages of MCED screening, the success of implementation is strongly dependent on patient education, trust in healthcare providers and screening techniques, and the ability to make the right decision in each case.

Cancer screening aims to identify a malignancy earlier that could lead to a less advanced and more treatable stage, resulting in reduced morbidity and mortality rates [3, 6]. Existing screening tools that have been demonstrated to be efficacious are not optimal, and their population-level effectiveness is often limited by continuing, well-described barriers [7, 8]. Low uptake rates are an ongoing challenge, as evidenced by the failure of colorectal cancer screening to reach national targets in many countries, despite the availability of multiple effective test options [8]. Such differences can be even greater for racial/ethnic minorities, lower socioeconomic status (SES), and rural populations, widening the gap in cancer outcomes [9]. In addition, structural barriers like medical mistrust, a product of historical evils and contemporary marginalization, can discourage individuals from accessing preventive health care [10, 11]. The picture is further muddied by recommendations for, or against, cancer screening that are changed commonly and occasionally made even in contradiction to the direction of expert bodies, which can sow confusion among both lay communities and front-line health workers, resulting in inertia and procrastination [3, 12]. A patient who tries to navigate through this labyrinth has to consider systemic and logistical factors in addition to personal beliefs and situation. Nevertheless, screening decisions are shaped not only by the effectiveness of their outcomes and results, but also by how individuals perceive their values [13].

The construct of perceived value in health behavior can be described as an individual’s assessment of the benefits of a certain health action in relation to its costs or barriers and has been integrated into theories of health behavior, such as the health belief model [14, 15]. A large body of evidence also supports that this perceived risk is an influential factor in screening behaviors for various types of cancer [13, 16]. For instance, women who highly valued mammography in detecting cancer early were much more likely to attend for regular screening (a 40% relative uptake increase compared with low perceived value) [13, 16]. Similarly, in previous studies of colorectal cancer screening, participants with a stronger perceived benefit of colonoscopy in preventing cancer were more likely to overcome barriers such as embarrassment and bowel preparation, which led to improvement in completion rates from 52 to 78% among average-risk adults [16, 17]. This relationship has also been quantified in a meta-analysis that found that higher perceived value was associated with a two- to three-fold greater likelihood of screening uptake for breast, cervical, and colorectal cancers [13]. Overall, these findings show that value is not an empty concept but a real predictor of psychological reactions to tests and predicts actual testing behavior at least in part by bridging the gap between test availability and uptake in the population. Hence, it is essential to learn how new technologies are valued to estimate their uptake in the real world.

Application of traditional single-organ cancer screening tests has been extensively studied in a large research literature regarding psychosocial and behavioral predictors, yet a relatively small amount of work has examined MCED tests [16, 18, 19]. Specifically, population-level knowledge about the perceived importance of this novel screening tool by the general public and its determinants is scarce [19, 20]. To date, studies have mostly measured awareness or intention to undergo MCED testing [21–23]. For instance, new polling evidence has suggested both low awareness (about 15–20%) and high support of equal to or greater than 60% in response to the question whether respondents would accept an MCED blood test (without delving into determinants or reasons or estimating value driving endorsement) [21, 23].

Furthermore, most existing studies have focused on demographic characteristics or individual predictors in isolation and have not examined the broader behavioral mechanisms underlying perceived value using an established theoretical framework. Consequently, relatively little is known about how cognitive beliefs, trust in science and healthcare, health information-seeking behaviors, digital access, healthcare experiences, and social influences collectively contribute to perceptions of MCED testing. Understanding these determinants is particularly important because perceived value may influence future discussions with healthcare providers, willingness to consider emerging screening technologies, and eventual adoption once MCED testing becomes more widely available. To address these gaps, the present study applies the PRECEDE–PROCEED framework to systematically examine predisposing, enabling, and reinforcing factors associated with perceived value of MCED testing among a nationally representative sample of U.S. adults. Unlike previous studies that have primarily described awareness or willingness to undergo MCED testing, this study adopts a theory-driven approach to investigate the behavioral determinants of perceived value while integrating survey-weighted inferential analyses with predictive machine learning methods. Specifically, this study aims to (1) characterize levels of perceived value of MCED testing among U.S. adults; (2) examine the demographic, clinical, and psychosocial correlates associated with perceived value; and (3) investigate the association between perceived value and individuals’ intentions to discuss MCED testing with their primary care providers.

### Conceptual framework and hypotheses

#### PRECEDE–PROCEED framework

To examine these questions, we apply the PRECEDE–PROCEED framework to organize candidate determinants and guide hypothesis development, a widely used model for predicting and evaluating health behavior and public health interventions, to explore the determinants of perceived value of multi-cancer early detection (MCED) testing [24, 25]. The model begins with desired health outcomes and works backward to identify the behavioral and environmental factors relevant to those outcomes. The PRECEDE part of the framework focuses on finding behavior determinants before creating models. Specifically, it separates predictors into three groups: predisposing, enabling, and reinforcing factors; together, they explain why people do or don’t participate in health behaviors.

#### Predisposing factors

These are individual-level cognitive and psychological factors that drive or hinder health behavior before actors even take action. These characteristics combine beliefs, knowledge, attitudes and perceptions of risk as well as orientations toward health information and medical science in general [15, 24]. In the cancer screening domain, predisposing factors often go on to determine whether or not individuals believe a given screening technology is advantageous, trustworthy, or relevant to their lives [13, 16].

Previous studies show that trust in scientific institutions, health care providers and public health authorities is an important determinant of attitudes toward new medical technologies [10, 26]. New tests will be trusted and valued more by those individuals who believe and trust in scientific and medical institutions. Likewise, proactive health information-seeking behaviors likely reflect engagement with health information that facilitates awareness and perceived usefulness of emerging screening tools. Knowledge and risk literacy also affect how people make health decisions because they influence people’s ability to interpret their cancer risks and their understanding of the potential benefits of early detection [13].

Based on these theoretical considerations, we propose the following hypotheses:

##### H1a

Higher trust in science is associated with higher perceived value of MCED testing.

##### H1b

Cancer information seeking is associated with higher perceived value of MCED testing.

##### H1c

Greater cancer knowledge and risk numeracy are associated with higher perceived value of MCED testing.

##### H1d

Having a family history of cancer is associated with higher perceived value of MCED testing.

##### H1e

Cancer-related beliefs (e.g., fatalistic beliefs and perceived cancer risk) are associated with perceived value of MCED testing.

#### Enabling factors

Enabling factors are structural or resource-based conditions that support (or inhibit) health behaviors. Access to health services, technology, insurance, and health information are some of these factors. Whereas predisposing factors give rise to motivation, enabling factors refer to practical capacity and whether individuals can follow through with that motivation [24, 27].

Access to health information and healthcare structure provide positive determinants, directly or indirectly influencing perceived value in MCED testing. Frequent users of digital technologies, seekers of online health information and people who engage with digital health resources are likely to be aware of novel screening technology and therefore more likely to view it as valuable. Another possible factor that influences people find messages about new screening tests is digital access (e.g., use of a smartphone, engagement with the internet) [28, 29].

Factors within the health care system may also influence perceived value. The extent to which individuals feel that screening technologies are overseen by the healthcare system, its quality and their own experiences with providers may also affect perceived legitimacy [26]. In contrast, negative encounters in healthcare settings (e.g., discrimination of any sort or the perception that care is a struggle) may reduce trust and therefore perceived value for new medical technologies [10].

Accordingly, the following hypotheses are proposed on this theoretical basis:

##### H2a

Greater engagement with digital health information (e.g., online health information seeking and smartphone access) will be associated with higher perceived value of MCED testing.

##### H2b

Individuals with higher trust in the healthcare system will report higher perceived value of MCED testing.

##### H2c

Structural barriers within healthcare systems, such as experiences of discrimination in medical care, will be associated with lower perceived value of MCED testing.

#### Reinforcing factors

Reinforcing factors are social and interpersonal influences that support or maintain general health attitudes and behaviors after the initial motivation is formed. These influences include recommendations from family members, the interaction between patients and health care providers, and social support regarding health-related decision-making [24, 30]. In the context of cancer screening, provider communication is an especially critical reinforcing mechanism. Patients who perceive themselves as supported by healthcare providers, are given opportunities to ask questions, and receive explanatory information about medical decision-making in general may be empowered with stronger confidence in screening technologies [16, 31]. And even interpersonal experiences, such as personal and family cancer history, can prime increased perceived relevance of early detection technologies by making the risk for cancer more personally salient [16].

Within the PRECEDE–PROCEED framework, reinforcing factors therefore represent the social processes that sustain health attitudes and translate them into behavioral intentions [24]. In the context of MCED testing, these influences may shape both the perceived value of the technology and individuals’ willingness to discuss screening options with healthcare providers.

Accordingly, we propose the following hypothesis:

##### H3

Reinforcing social influences, including family cancer history and supportive healthcare interactions, will be associated with a higher perceived value of MCED testing.

#### Controls

Variables include age, education, household income, financial comfort, race, and marital status, which are added to take into account sociodemographic differences beyond expectations, which may influence perceived barriers to and facilitators of MCED testing but will not be the main behavioral determinants in the PRECEDE–PROCEED framework. Adjusting for these factors allows us to isolate the relationships between predisposing, enabling, and reinforcing variables on the perceived value of MCED testing. Since the perceived value of MCED testing may reflect sociocultural and socioeconomic differences, these factors are treated as covariates (sociodemographic controls), to minimize confounding when estimating predictors.

## Methods

### Study design

We conducted a cross-sectional secondary data analysis using Cycle 7 of the Health Information National Trends Survey (HINTS 7). The HINTS database is a nationally representative survey of the US noninstitutionalized adult population [32]. The purpose of the survey is to investigate the American population’s needs in terms of health-related information and their access to care [33, 34]. The sampling strategy for the HINTS survey consists of a two-stage design. In the first stage, a stratified sample of addresses is selected from a file of residential addresses [35]. In the second stage, one adult is selected within each sampled household. Respondents are offered the choice to respond online or through a paper survey [35]. Both modes of the survey (paper and online) were offered in English or Spanish [35]. All groups received a $2 pre-paid monetary incentive to encourage participation [35]. Respondents in the control group were offered a bonus incentive to complete the survey online. Returned surveys were reviewed for completion and duplication (more than one questionnaire returned from the same household) to ensure they were eligible for inclusion in the final dataset [35]. Further details on survey design and sampling strategies are published elsewhere [32]. All analyses incorporated the full-sample person-level weight to generate population-relevant descriptive estimates.

### Measures

**Fig. 1:**
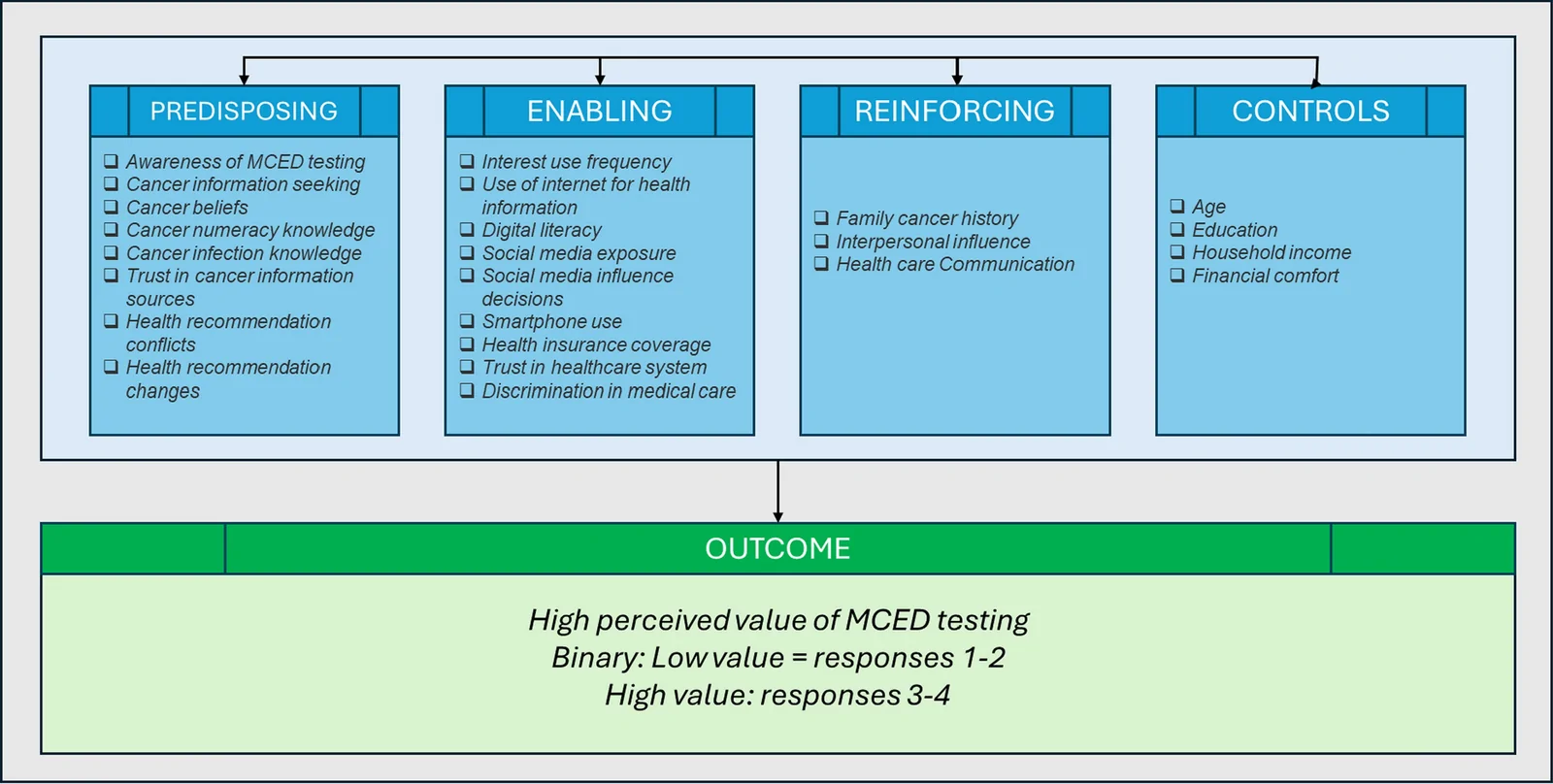
The PRECEDE-based variables

Candidate variables from the HINTS survey related to predictors of perceived value of multi-cancer early detection (MCED) testing were selected a priori and mapped to these domains based on theory rather than data-driven selection. HINTS Cycle 7 included two separate MCED-related survey items. Awareness of MCED testing (Question 4) was treated as a predisposing factor within the PRECEDE framework, whereas perceived value of MCED testing (Question 5) served as the primary outcome variable for this study. Figure 1 illustrates the conceptual model linking PRECEDE determinants to the perceived value of MCED testing.

### Data analysis

**Fig. 2:**
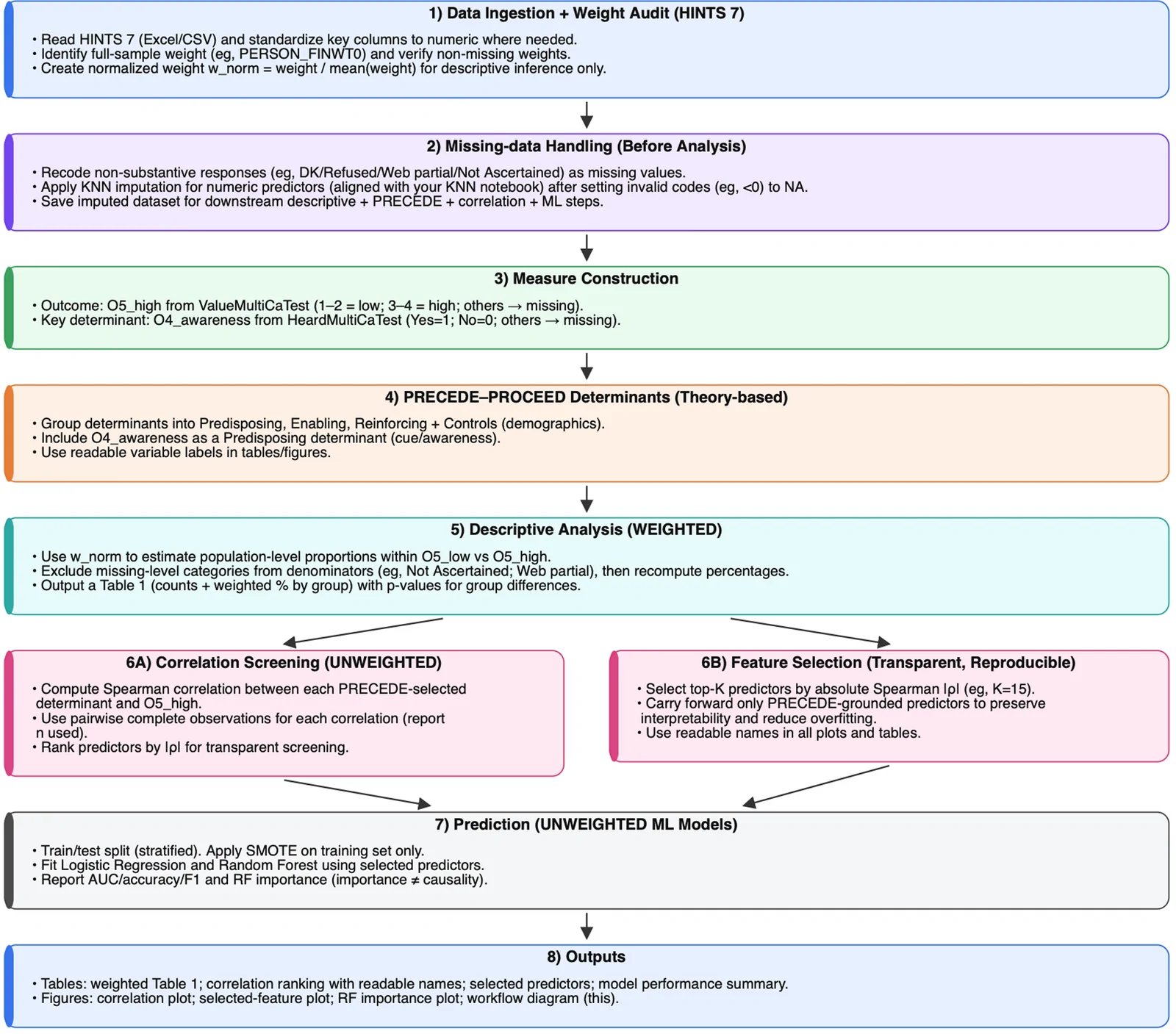
Methods workflow for this study

To provide nationally representative descriptive estimates, we used the HINTS person-level full-sample weight (*PERSON_FINWTO*). To improve numerical stability, weights were normalized by dividing each respondent’s weight by the mean weight among respondents with non-missing weights. Weighted proportions were used to estimate the population distribution of perceived MCED value and demographic characteristics. For outcome-stratified descriptive comparisons, weighted percentages were calculated separately for low and high perceived value groups. Analyses proceeded in four structured stages aligned with the research questions. A multiple imputation method was used to handle missing data. Multiple imputations are useful for handling missing data problems and accounting for imputation uncertainty [36, 37]. The K-nearest-neighbor algorithm method is used for that, where the missing values get replaced by the nearest neighbor estimated values [37]. All analyses were conducted in Python using standard statistical and machine learning libraries. Weighted descriptive statistics were computed using survey-adjusted procedures (See Fig. 2).

### Study population and analytic sample

The sample size of the HINTS 7 database comprised 7,278 respondents. The dataset was reduced to 6,728 (92.4%) participants for analysis after screening for missing responses for the main outcome of interest (perceived value of MCED testing). The remaining 550 (7.6%) respondents were excluded due to missing values for the main outcome of interest. The missing observations for other predictor variables were managed using the *k*-nearest neighbors (KNN) algorithm (*k*=3) with an aim to maximize the number of participants used for statistical analysis.

### Weighted descriptive analysis

First, weighted proportions were calculated to estimate the population distribution of perceived MCED value (Q1). Outcome-stratified descriptive tables compared demographic characteristics between high and low perceived value groups.

### Correlation screening

Second, Spearman rank correlations were calculated between each PRECEDE-collapsed determinant and O5_high. Spearman’s *ρ* was selected due to binary predictors and non-normal distributions. Predictors were ranked by magnitude of association to identify high-signal determinants.

### Machine learning prediction models

Finally, to address the last research question, two supervised classification models were trained to predict O5_high using only theory-based PRECEDE determinants: Logistic Regression and Random Forest. Machine learning models were used to complement the results of the survey-weighted logistic regression by testing if the same set of predictors would be associated with the outcome in other prediction methods.

To provide both inferential and predictive perspectives, this study combined survey-weighted logistic regression with supervised machine learning models. Survey-weighted logistic regression was used to identify independent factors associated with perceived value of MCED testing while accounting for the complex sampling design of HINTS 7. Machine learning models were subsequently applied to evaluate the relative predictive importance of the selected variables and assess the consistency of predictor rankings across analytical approaches.

Survey-weighted logistic regression models were used to estimate the association between PRECEDE-based determinants and high perceived value of MCED testing. The dependent variable, the binary outcome that representing the perceived value of MCED, was modeled using the logit link function; PRECEDE predictors were included as independent variables and sociodemographic characteristics (age, gender, race/ethnicity, education, income, and marital status) were included as covariates to control for confounding. Model parameters were estimated by means of maximum likelihood estimation with incorporation of survey weights to adjust for the complex sampling design. Results were presented as adjusted odds ratios (ORs) with 95% confidence intervals (CIs), and statistical significance was assessed at *α* = 0.05.

In addition to the inferential framework, supervised machine learning models were applied to evaluate predictive performance. Classification models including logistic regression as a linear baseline classifier and ensemble tree-based methods (e.g., Random Forest) to capture nonlinear relationships. The interaction effect was trained on the imputed and preprocessed dataset. The data were then stratified sampled, such that this dataset was split into a training (70%) and testing (30%) set, to ensure that class distribution was preserved. We further used k-fold cross-validation on the training set to ensure the robustness of our model. We then tested the performance of our prediction algorithms across multiple evaluation metrics: There are accuracy, precision, recall (sensitivity), F1-score and area under receiver operating characteristic curve (AUC–ROC), which provided a comprehensive assessment of discrimination and classification performance.

## Results

### Demographic characteristics of the sample

Among 7,278 participants, 550 samples are missing, and 92.4% (*n* = 6,728) of the complete cases were used for analysis. 74.9% of the participants (*n* = 5,037) reported perceiving high value of MCED, and 25.1% of the participants (*n* = 1,691) reported perceiving low value of MCED. Most of the participants are White (60.6%), followed by Hispanic (17.5%) and Black (11.0%). The majority of the participants have been to some colleges (38.6%) and are college graduates (34.0%).

### MCED perceived value across demographics

Through weighed descriptive analysis, significant differences were found in MCED perceived value across age, education, race, income and financial status, marital status, and healthcare experience. Younger groups (18–39) had more frequent reports of low perceived value (45.9%), while middle-aged groups (40–59) showed higher high perceived values proportions (39.5%) indicating increasing acceptance with age. Higher educational level perceived to be more valuable. College graduates made up a greater proportion of the high-value group (35.3%) than the low-value group (30.4%). Low proportions in the high-value group and relatively higher proportions in the low-value group were observed among Black and Hispanic participants compared to the overall population. Individuals with higher income overall had the higher perceived value MCED. Married individuals were more likely to report high MCED value (59.8%) compared to unmarried individuals. Additionally, individuals reporting experiences of discrimination in healthcare were disproportionately represented in the high-value group, suggesting complex relationships between prior experiences and perceived benefit of new screening technologies.

Most participants (74.9%) reported perceived high value of MCED, and demographic differences were found in perceived value of MCED. People who are reported as 40 to 59 years old (39.5%, *p*<0.001), White (60.3%, *p*<0.001), household income ranges from $100,000 to $199,999 (23.7%, *p*<0.001), married (59.8%, *p*<0.001), and did not experienced discrimination in medical care (82.8%, *p*<0.001) are more likely to perceive high value of MCED. More details can be found in Appendix Table 1.

**Table 1:** Spearman correlation between PRECEDE determinants and high perceived MCED value (O5_high)

PRECEDE category	Predictor variable	Spearman *ρ*	Rank
Predisposing	Trust in science	0.101	1
	Cancer information seeking	0.1	2
	Cancer fatalism (agree)	0.091	3
	Trust in government	0.082	6
	Trust in physician	0.072	8
	Knowledge that HBV causes cancer	0.061	9
	Knowledge in cancer risk (Numeracy)	0.058	10
	Too many cancer recommendations (agree)	0.057	12
	“Everything causes cancer” belief	0.054	13
	Knowledge that HCV causes cancer	0.049	14
	MCED awareness (O4)	−0.012	>15
	Conflicting recommendations (high)	NA	>15
	Changing recommendations (high)	NA	>15
Enabling	Trust in healthcare system	0.086	4
	Online health information use	0.083	5
	Smartphone access	0.058	11
	Experienced discrimination in care	0.04	15
	Health insurance coverage	0.022	>15
	Internet use frequency	NA	>15
	Digital literacy (high)	NA	>15
	Social media exposure (visited)	NA	>15
	Social media exposure (watched video)	NA	>15
	Social media influences decisions (high)	NA	>15
	Frequency of provider visits	NA	>15
	Perceived quality of care (high)	NA	>15
Reinforcing	Family cancer history	0.078	7
	Family support/trust (high)	0.038	>15
	Chance to ask questions (high)	NA	>15
	Involvement in decisions (high)	NA	>15
	Clear explanations (high)	NA	>15
	Help managing uncertainty (high)	NA	>15

### Predisposing, enabling, and reinforcing factors associated with MCED perceptions

The variables are selected by using PRECEDE method. Most selected variables are predisposing factors, relating to trust (science, physicians, government), cancer knowledge (HBV/HCV awareness, numeracy), beliefs (fatalism, generalized cancer beliefs), and information-seeking behaviors. See more details in Appendix Table 2.

**Table 2:** Outcome O5: perceived value of MCED (O5_value 1–4)

O5_value level	Meaning	*n*	% of total (*n*=7,278)	Interpretation
4	Very valuable	3,013	41.4%	Largest group rates MCED as very valuable
3	Somewhat valuable	2,024	27.8%	Moderate support is common
2	A little valuable	1,105	15.2%	Smaller group shows weak endorsement
1	Not at all valuable	586	8.1%	Strong rejection is minority
Missing	Missing (no response)	550	7.6%	Excluded from outcome-based analyses

Correlation analysis with Spearman coefficients showed that several PRECEDE-based determinants were positively associated with high perceived MCED value, as summarized in Table 1. The top 15 PRECEDE variables ranked by absolute spearman correlation are selected for later analyses.

The strongest correlations identified were trust in science (*ρ* = 0.101) and cancer information-seeking behavior (*ρ* = 0.100), followed by cancer fatalistic beliefs (*ρ* = 0.091), and trust in the healthcare system (*ρ* = 0.086). Other variables that were strongly and positively related to MCED valuation included receiving health information online (*ρ*= 0.083), trust in government(*ρ*=0.082), and family history of cancer(*ρ*=0.078).

Other factors, such as trust in physicians, knowledge of cancer risk factors (HBV and HCV), and cancer risk numeracy, showed smaller but still positive correlations. This influence of various individual factors points more toward a diverse interplay rather than any single measurement from our experiments being the sole predictor for MCED perception.

### Predictors of the MCED perceptions

As shown in Table 2, the original dataset O5 has 4 different levels, and it is converted to binary variable, as demonstrated in Table 3.

**Table 3:** Binary outcome used in analyses: O5_high (low=1–2, high=3–4)

Category	*n*	% among non-missing (*n*=6,728)	Interpretation
High value (3–4) = 1	5,037	74.9%	About 3 in 4 rate MCED as high value
Low value (1–2) = 0	1,691	25.1%	About 1 in 4 rate MCED as low value
Missing	550	/	Dropped for correlation/ML

Multivariable logistic regression analysis revealed that all selected PRECEDE-based predictors were significantly associated with high perceived MCED value (all *p* < 0.001), as shown in Table 4.

**Table 4:** Odds ratios for high MCED value

PRECEDE category	Predictor variable	Category	OR (95% CI)	*p*-value
Predisposing	Trust in science	No	Reference	<0.001
		Yes	1.80 (1.56–2.07)	
	Cancer information seeking	No	Reference	<0.001
		Yes	1.59 (1.42–1.78)	
	Cancer fatalism (agree)	No	Reference	<0.001
		Yes	1.52 (1.36–1.70)	
	Trust in government	No	Reference	<0.001
		Yes	1.50 (1.33–1.69)	
	Trust in physician	No	Reference	<0.001
		Yes	1.89 (1.52–2.33)	
	Knowledge that HBV causes cancer	No	Reference	<0.001
		Yes	1.48 (1.27–1.72)	
	Cancer risk numeracy (correct)	No	Reference	<0.001
		Yes	1.33 (1.18–1.50)	
	Too many cancer recommendations (agree)	No	Reference	<0.001
		Yes	1.34 (1.19–1.51)	
	‘Everything causes cancer’ belief	No	Reference	<0.001
		Yes	1.30 (1.16–1.46)	
	Knowledge that HCV causes cancer	No	Reference	<0.001
		Yes	1.36 (1.17–1.59)	
Enabling	Trust in healthcare system	No	Reference	<0.001
		Yes	1.64 (1.43–1.88)	
	Online health information use	No	Reference	<0.001
		Yes	1.63 (1.41–1.87)	
	Smartphone access	No	Reference	<0.001
		Yes	1.54 (1.28–1.84)	
	Experienced discrimination in care	No	Reference	<0.001
		Yes	1.30 (1.11–1.52)	
Reinforcing	Family cancer history	No	Reference	<0.001
		Yes	1.45 (1.29–1.63)	

Trust-related variables showed the strongest associations of all predictors. Each point increase in trust was associated with 1.89 times the odds of perceiving MCED as valuable compared to those with lower trust (95% CI: 1.52–2.33). Likewise, trust in science (OR = 1.80, 95% CI: 1.56–2.07) and trust in the healthcare system (OR = 1.64, 95% CI: 1.43–1.88) were positively correlated with perceived value of the data at a high level or higher level of inclusion as well. Online health information use was also positively correlated (OR = 1.63, 95% CI: 1.41–1.87). Sociobehavioral and experiential factors were also moderately associated.

People with higher odds of high perceived value included those actively seeking cancer-related information (OR = 1.59), with access to a smartphone (OR = 1.54), and having fatalistic views toward cancer (OR = 1.52). In addition, trust to government (OR = 1.50), knowledge on hepatitis B as a risk factor of cancer (OR = 1.48), and family history of cancer (OR = 1.45) were all positively associated with perceived value.

Other predictors demonstrated more modest but significant effects: knowledge of hepatitis C (OR = 1.36), perception of excessive cancer recommendations (OR = 1.34), cancer risk numeracy (OR = 1.33), generalized cancer beliefs (OR = 1.30), and experiences of discrimination in care (OR = 1.30).

Predisposing factors were the largest contributor of any significant predictor across PRECEDE domains, followed by enabling factors with little contribution from reinforcing factors.

To complement the inferential analyses, machine learning models were applied to evaluate the relative predictive importance of the selected variables. The predictor rankings were generally consistent with the regression analyses, with trust-related variables and health information engagement emerging as the strongest contributors to model performance. More details of the model performance can be found in Appendix Table 4.

## Discussion

This study aimed to identify the factors influencing perceived value of multi-cancer early detection (MCED) testing through a theory-driven approach. Overall, the findings support the application of the PRECEDE–PROCEED framework for understanding determinants perceived value of MCED testing. Instead of treating each one as a singular determinant, this pattern is about building value through interaction of psychological, structural, and social influences.

Although performing additional diagnostic tests such as multicollinearity assessment and sensitivity analysis can provide more information about the model stability, the consistency of the major predictors identified across both inferential and machine learning approaches provides additional support for the robustness of the observed associations.

The findings indicate a hierarchy of influence, where predisposing variables (trust, beliefs, and knowledge) lead to the initial formations of attitudes, enabling factors (encompassing, digital access and engagement; trust in the system) enable awareness and understanding, and reinforcing factors (social and clinical interactions) maintain attitudes and reinforce behavioral intentions. These findings also suggest that perceived value of MCED testing is overall high among U.S. adults, but not uniformly distributed. Rather, it is shaped by interplays of psychological trust factors, information engagement behaviors, and structural access mechanisms within the framework, where trust-related factors remain as the most consistent and impactful correlates.

### Predisposing factors: the central role of trust and beliefs

Across all domains, trust in physicians and science as well as cancer information-seeking behavior, strongly predicted perceived value of MCED testing. These results highlight the role of psychosocial and cognitive factors on willingness to adopt such novel health technologies.

Trust is recognized as a foundational component of health decision-making, especially when uncertainty or new interventions involved. Previous work has suggested that acceptance of medical recommendations and screening behaviors strongly correlate with trust in scientific institutions or healthcare providers [10, 26]. The current results expand on this literature by showing that trust is not only an important factor in existing screening methods but also a key determinant of perceived value for new technology platforms such as MCED.

The next most significant factor was cancer information seeking, that is, individuals who actively searched for cancer-related information stand a higher chance of appreciating the potential benefits arising from early detection technologies. This finding aligns with previous research indicating that individuals who seek cancer-related information are more likely to engage in prevention and screening [38].

Other cancer-related beliefs such as fatalism and general perceptions of getting cancer were also associated with perceived value. Even though fatalism has generally been seen as an obstacle needed to be overcome for screening, other research indicates that at least in certain contexts, increased worry regarding cancer outcomes may enhance early detection motivation [39]. The current findings indicate that those preexisting beliefs should have an effect on how people predict the value of MCED technologies.

### Enabling factors: the role of digital access and healthcare infrastructure

Enabling factors, especially trust in the healthcare system, online health information use, and digital access (e.g., smartphone use) were also significant predictors of perceived MCED value. Such insights highlight the role of structural and informational access in influencing attitudes toward new screening technologies.

The effects of digital health engagement on perceived value are substantial, implying that those who are more connected to their own health information systems are more likely to come across MCED as a new technology and be able to understand its existence within them. This is consistent with earlier studies that indicated that Internet access to health information and e-health tools predicted higher awareness levels of preventive health behaviors [28, 29].

Another crucial enabling factor mentioned by the participants was trust in the health system, confirming the notion that users need to have faith not only in technology itself but also in an entity responsible for its provision. Trust in the healthcare system has been found to relate to use of preventive services and adherence to recommended medical treatment [26].

On the other hand, experiences of discrimination or barriers in healthcare settings can lead to a diminished perceived value due to loss of trust. While generally smaller, these effects are consistent with the literature showing that negative experiences with healthcare may discourage engagement with preventive services [10].

### Reinforcer factors: the impact of social and clinical environment

Although the present study focused on PRECEDE framework constructs, these factors do not exist in isolation. Consideration of broader social determinants of health is critical when implementing interventions at the community level, including socioeconomic status, geographic location, educational resources, and access to high-quality care. These factors, among others, may intersect with or influence patients’ perceptions of the value of health information, their interactions with healthcare providers, and the degree to which they can trust those providers. Thus, attention to these factors is needed to ensure that the promise of MCED technologies can be realized by all patients.

Reinforcing factors such as family cancer history and experience interacting with the healthcare system also contribute to perceived value. Children of family members with cancer probably experience increased perceptions of susceptibility, resulting in increased personal relevance regarding possible benefits of early detection. This finding is consistent with previous studies that found family history of cancer to be positively associated with engagement in screening behaviors [16].

The healthcare interaction, especially communication with providers, is a critical reinforcing mechanism. Communicating these data can build confidence in medical technologies as well as incentivize patients to discuss screening opportunities. Previous research has shown that patient–provider communication is an important predictor of screening uptake [31].

While they might not be the main correlates of perceived value, reinforcing factors are key to maintaining and enacting attitudes. Interventions promoting MCED should therefore encourage provider–patient discussion, enhance communication quality in clinical settings, and leverage family-based risk communication.

The present results should also be interpreted within the broader literature on MCED testing. Although these tests represent an attractive opportunity for improving early cancer detection, questions remain in terms of clinical utility, downstream diagnostic pathways, cost-effectiveness, and the actionability of positive test results across different healthcare settings. Therefore, understanding the factors associated with perceived value is therefore particularly important during this period of rapid technological development, as public acceptance may be influenced not only by scientific evidence but also by communication regarding the benefits, limitations, and uncertainties of MCED testing.

### Implications for policy and practice

There are several important implications of the findings for MCED testing implementation. First, strengthening the public confidence in science and people providing care can be essential for the acceptance of new screening technologies. Second, digital health communication can play an important role in increasing awareness and understanding of preventative primary care or healthcare options. Third, adoption must be equitable, which requires attention to structural barriers and differences in experiences within healthcare. Last, provider conversations around patient uptake may aid the transition from perceived value to actual uptake.

#### Limitations

The findings of this study should be interpreted under the following limitations. First, the analysis used cross-sectional, self-reported survey data from the HINTS dataset. Data from self-reported responses are inherently subject to recall bias, social desirability bias, and variability in respondents’ interpretation of questions about perceptions, beliefs, and behavioral intentions pertaining to multi-cancer early detection (MCED) testing. Furthermore, the cross-sectional data limit causal inferences between PRECEDE framework factors and preferences for MCED tests.

Second, the outcome variable is represented as binary (yes or no) for easy interpretability in predictive modeling and analytic framework compliance. While this strategy simplified analysis and interpretation of the model, it may have sacrificed some resolution in participant responses (i.e., likelihood of acceptance, hesitancy or perceived value), potentially masking more subtle differences in acceptance-related measures. Future studies may benefit from retaining ordinal response formats where appropriate or application of multiclass modeling methods to capture heterogeneity in responses.

Third, although to mitigate the problem of missing data on numeric covariates K-nearest neighbor (KNN) imputation was used, which presumes that observations with similar patterns in the features can reliably predict each other’s missing values (observation A should be able to accurately provide the value of observation B given that they share a feature pattern). Additionally, categorical and text-encoded survey responses could not be fully utilized in the imputation step without leaving residuals of missingness after preprocessing.

In addition, HINTS does not capture several implementation-specific factors that have the potential to influence perceptions of MCED testing (e.g., anticipated cost of the test, financial burden of follow-up diagnostic evaluations after a positive result, and uncertainty regarding appropriate clinical management following positive or indeterminate findings). As MCED technologies move toward broader clinical implementation, these practical considerations may substantially influence perceived value and adoption. Future research should examine how these factors affect acceptance and decision-making regarding MCED testing.

Finally, since the MCED technologies that we ask about are still in early stages of cancer screening innovation, participant statements may reflect a hypothetical mindset rather than an actual behavioral intention to adopt. While awareness and understanding of MCED testing is evolving, survey-reported preferences may not translate to actual clinical decision-making once these technologies are more fully implemented. Furthermore, although survey-weighted logistic regression and machine learning models were used to provide complementary inferential and predictive perspectives, the relative importance of individual predictors may vary across different study populations and analytical approaches. Consequently, the findings should be interpreted within the context of the present dataset, and future studies using independent datasets and longitudinal designs are needed to further evaluate the stability and generalizability of the identified associations.

## Conclusions

All in all, this study shows that perceived value of MCED testing is influenced mainly by trust and information engagement, access-related factors, while reinforcers may play a complementary role. This research represents the first systematic investigation into behavioral mechanisms of acceptance about new cancer screening technologies and lays the groundwork for carefully constructed interventions targeted on real-world adoption of these life-saving techniques.

## Data Availability

The data that support the findings of this study are publicly available from the National Cancer Institute Health Information National Trends Survey (HINTS) program. The datasets can be accessed at https://hints.cancer.gov.

## Appendix

See Tables 5, 6, 7, 8.

**Table 5:** Weighted descriptive analysis table

Category	O5_low (1–2)	O5_high (3–4)	All	*p*_value
Age				
18–39	506 (45.9%)	1039 (29.5%)	1545 (33.8%)	0.000
40–59	378 (25.6%)	1517 (39.5%)	1895 (35.8%)	
60–79	600 (22.3%)	2078 (26.9%)	2678 (25.7%)	
≥80	168 (6.2%)	326 (4.2%)	494 (4.7%)	
Education				
Less than high school	123 (8.5%)	307 (5.2%)	430 (6.1%)	0.000
High school	325 (23.7%)	783 (20.5%)	1108 (21.3%)	
Some college	464 (37.4%)	1465 (39.1%)	1929 (38.6%)	
College graduate	754 (30.4%)	2418 (35.3%)	3172 (34.0%)	
Race				
White	973 (61.5%)	2555 (60.3%)	3528 (60.6%)	0.000
Black	191 (9.2%)	769 (11.7%)	960 (11.0%)	
Hispanic	272 (16.1%)	1042 (18.0%)	1314 (17.5%)	
Asian	88 (6.8%)	252 (5.1%)	340 (5.6%)	
Other	70 (6.3%)	184 (4.9%)	254 (5.3%)	
Hispanic origin				
Cuban only	14.5 (0.8%)	57.0 (1.2%)	71.5 (1.1%)	0.000
Mexican only	150.5 (8.6%)	356.7 (7.3%)	507.2 (7.7%)	
Multiple Hispanic ethnicities selected	22.4 (1.3%)	41.4 (0.9%)	63.8 (1.0%)	
Not Hispanic only	1462.7 (84.0%)	3997.3 (82.1%)	5460.0 (82.6%)	
Other Hispanic only	76.5 (4.4%)	305.3 (6.3%)	381.9 (5.8%)	
Puerto Rican only	15.3 (0.9%)	108.3 (2.2%)	123.6 (1.9%)	
Household income				
$0 to $9,999	164 (11.5%)	321 (6.0%)	485 (7.5%)	0.000
$10,000 to $14,999	82 (4.0%)	232 (3.8%)	314 (3.9%)	
$100,000 to $199,999	291 (23.1%)	1008 (23.7%)	1299 (23.5%)	
$15,000 to $19,999	84 (5.5%)	219 (2.9%)	303 (3.6%)	
$20,000 to $34,999	203 (12.1%)	593 (11.4%)	796 (11.6%)	
$200,000 or more	126 (8.4%)	427 (11.0%)	553 (10.3%)	
$35,000 to $49,999	205 (12.3%)	570 (11.3%)	775 (11.6%)	
$50,000 to $74,999	244 (13.8%)	782 (16.3%)	1026 (15.6%)	
$75,000 to $99,999	170 (9.4%)	612 (13.5%)	782 (12.4%)	
Financial comfort				
Finding it difficult on present income	250 (16.4%)	852 (19.2%)	1102 (18.4%)	0.000
Finding it very difficult on present income	133 (8.8%)	415 (8.3%)	548 (8.5%)	
Getting by on present income	596 (36.6%)	1870 (37.9%)	2466 (37.6%)	
Living comfortably on present income	672 (38.1%)	1764 (34.4%)	2436 (35.4%)	
Multiple responses selected in error	2 (0.1%)	11 (0.1%)	13 (0.1%)	
Marital status				
Married	781 (47.6%)	2592 (59.8%)	3373 (56.6%)	0.000
Not married	884 (52.4%)	2385 (40.2%)	3269 (43.4%)	
Experienced discrimination in medical care				
No	1438 (87.6%)	4119 (82.8%)	5557 (84.1%)	0.000
Yes	239 (12.4%)	887 (17.2%)	1126 (15.9%)	

**Table 6:** PRECEDE variable

Predictor Variable	PRECEDE Domain	Internal Variable Name	Type	Operational Definition
Trust in science	Predisposing Factors	PRE_A_trust_science	Binary	High vs. low trust in scientists for cancer information
Cancer information seeking	Predisposing Factors	PRE_A_seek_info	Binary	Ever actively sought cancer-related information
Trust in physician	Predisposing Factors	PRE_A_trust_doc	Binary	High vs. low trust in doctors
Trust in government	Predisposing Factors	PRE_A_trust_gov	Binary	High vs. low trust in government health information
Conflicting health recommendations	Predisposing Factors	PRE_A_recs_conflict_high	Binary	High vs. low perceived conflict in recommendations
Changing health recommendations	Predisposing Factors	PRE_A_recs_change_high	Binary	High vs. low frequency of changing recommendations
Cancer fatalism (agree)	Predisposing Factors	PRE_P_cancer_fatalistic_agree	Binary	Agreement that cancer outcomes are unavoidable
Too many cancer recommendations (agree)	Predisposing Factors	PRE_P_too_many_recs_agree	Binary	Agreement that there are too many screening recommendations
“Everything causes cancer” belief	Predisposing Factors	PRE_P_everything_causes_cancer_agree	Binary	Generalized belief about cancer causation
Knowledge that HBV causes cancer	Predisposing Factors	PRE_O_hbv_knows_causes_cancer	Binary	Correctly identified hepatitis B as a cancer risk
Knowledge that HCV causes cancer	Predisposing Factors	PRE_O_hcv_knows_causes_cancer	Binary	Correctly identified hepatitis C as a cancer risk
Cancer risk numeracy (correct)	Predisposing Factors	PRE_P_numeracy_correct	Binary	Correct interpretation of a “1 in 100” risk
Family cancer history	Predisposing Factors	PRE_Q_family_history_any	Binary	Any immediate family member with cancer
MCED awareness (O4)	Predisposing Factors	PRE_O4_awareness	Binary	Prior awareness of MCED testing
Internet use frequency	Enabling Factors	PRE_B_internet_3cat	Ternary	Low/Medium/High internet use
Digital literacy (high)	Enabling Factors	PRE_B_diglit_high	Binary	High digital search skills
Smartphone access	Enabling Factors	PRE_B_smartphone	Binary	Uses a smartphone device
Online health information use	Enabling Factors	PRE_B_online_healthinfo	Binary	Used electronic sources for health information
Social media exposure (visited)	Enabling Factors	PRE_B_socmed_visit	Ternary	Frequency of visiting social media
Social media exposure (video)	Enabling Factors	PRE_B_socmed_video	Ternary	Frequency of watching health videos
Social media influences decisions	Enabling Factors	PRE_B_socmed_decisions_high	Binary	High influence of social media on decisions
Health insurance coverage	Enabling Factors	PRE_C_insured	Binary	Currently insured
Frequency of provider visits	Enabling Factors	PRE_C_freq_visit	Continuous	Number of healthcare visits
Perceived quality of care (high)	Enabling Factors	PRE_C_qualitycare_hi	Binary	High perceived quality of care
Trust in healthcare system	Enabling Factors	PRE_C_trust_system_hi	Binary	High vs. low trust in healthcare system
Experienced discrimination in care	Enabling Factors	PRE_C_discrimination	Binary	Ever experienced discrimination in medical care
Chance to ask questions (high)	Reinforcing Factors	PRE_Rf_chance_questions_hi	Binary	High opportunity to ask questions
Involvement in decisions (high)	Reinforcing Factors	PRE_Rf_involved_decisions_hi	Binary	High involvement in care decisions
Clear explanations (high)	Reinforcing Factors	PRE_Rf_explained_clearly_hi	Binary	Provider explanations were clear
Help managing uncertainty (high)	Reinforcing Factors	PRE_Rf_help_uncertainty_hi	Binary	Providers helped manage uncertainty
Age, education, income, race/ethnicity, marital status	Controls	Multiple	Categorical	Included as sociodemographic controls

**Table 7:** Key findings by effect size

Effect size category	OR range	Predictors included
Strong effects (OR > 1.6)	1.80–1.89	• Trust in physician (1.89) • Trust in science (1.80) • Trust in healthcare system (1.64) • Online health info use (1.63)
Moderate effects (OR 1.4–1.6)	1.40–1.59	• Cancer info seeking (1.59) • Smartphone access (1.54) • Cancer fatalism (1.52) • Trust in government (1.50) • Knowledge HBV causes cancer (1.48) • Family cancer history (1.45)
Weak effects (OR < 1.4)	1.30–1.39	• Knowledge HCV causes cancer (1.36) • Too many recommendations (1.34) • Cancer risk numeracy (1.33) • ‘Everything causes cancer’ belief (1.30) • Experienced discrimination (1.30)

**Table 8:** Machine learning model performance

Logistic regression model	
Metric	Value
Sample size	6,728
Training/test split	70%/30%
Accuracy	59.40%
AUC (Area under ROC curve)	0.623
precision (high MCED)	81%
recall (high MCED)	60%
F1-score (high MCED)	0.69
Confusion matrix	
− True negatives	287
− False positives	220
− False negatives	599
− True positives	913

Random forest model	
Metric	Value
Accuracy	66.80%
AUC	0.537
Precision (high MCED)	77%
Recall (high MCED)	80%
F1-score (high MCED)	0.78
Confusion matrix	
− True negatives	138
− False positives	369
− False negatives	302
− True positives	1,210

## Code availability

The code used for data processing and analysis is available from the corresponding author upon reasonable request.
